# Comparison of Gene Expression Profiles in Human Germinal Vesicle Before and After Cytoplasmic Transfer From Mature Oocytes in Iranian Infertile Couples 

**Published:** 2016-06

**Authors:** Fatemeh Sadat Hoseini, Nasser Salsabili, Firoozeh Akbari-Asbagh, Reza Aflatoonian, Seyed Hamid Aghaee-Bakhtiari

**Affiliations:** 1 Women’s Hospital, Tehran University of Medical Sciences, Tehran, Iran; 2 Women’s Hospital AND Department of Physiotherapy, School of Rehabilitation, Tehran University of Medical Sciences, Tehran, Iran; 3 Reproductive Biomedicine Research Center, Royan Institute for Reproductive Biomedicine, ACECR, Tehran, Iran; 4 Department of Medical Biotechnology, School of Medicine, Mashhad University of Medical Sciences, Mashhad, Iran

**Keywords:** Gene Expression, Cytoplasmic Transfer, Oocyte Maturity

## Abstract

**Objective:** To evaluate the effect of cytoplasm transfer from mature oocytes to germinal vesicle(GV)s on promoting the maturation of cytoplasm of GV at the mRNA level.

**Materials and methods:** Sixty six in vitro fertilization (IVF) operations between June 2012 and November 2013 were included in this study. Totally 120 GVs were obtained. Normal GVs were categorized into 3 groups (n = 40) randomly: the first comprised oocytes that did not receive the cytoplasm of mature oocytes; the second group comprised oocytes that did not receive the cytoplasm of mature oocytes but were incubated for 24 h; and the third group comprised oocytes that received 10-15% the cytoplasm of mature oocytes and were then incubated for 24 h. Each group was separately analyzed by quantitative polymerase chain reaction (qPCR) and the expression levels of selected genes were assessed.

**Results:** The expression levels of genes involved in the cytoplasmic maturity, and energy-producing mitochondria were significantly higher in the pooled oocytes of 2^nd^ control group than those of the 1^st^ control and intervention groups (p < 0.001). The genes involved in the meiosis, spindle check point, DNA repairing and cell cycle checkpoint did not have any expression in the 1^st^ and intervention groups; however, these genes were expressed in the 2^nd^ group, significantly. In the 2^nd^ group, the highest expression level was observed for genes involved in the DNA repairing and cell cycle checkpoint. In the intervention group, none of the genes were expressed except for energy-producing mitochondria gene; even in this case, the expression level of this gene in this group of oocytes was significantly lower than that in other groups (p < 0.001). After 24 h meiosis assumption was significantly higher in the third group than in the second group (95% vs. 68%, p < 0.001).

**Conclusion:** The cytoplasm transfer technique is not effective in cytoplasmic maturity of the recipient GV oocytes. In contrast, 24-hr in-vitro culture is associated with increased expression of studied genes in GVs.

## Introduction

The evolutionary quality of the oocyte and the embryo are of paramount importance in the success rate. The oocyte maturity depends on the maturity of both cytoplasm and nuclei. The expression patterns of the genes involved in the nuclei maturity are similar in mature in vitro and in vivo oocytes. However, the cytoplasm of in vitro oocytes remain immature. Conventional protocols, such as co-culture and improved culture, have not yet been able to overcome this shortcoming. In other words, the nuclei matures, but the cytoplasm does not improve (1). Impaired performance of the oocyte cytoplasm will lead to failure in fertilization, implantation, fetal development, and pregnancy. The expression of specific genes can be used as markers of oocyte quality. However, due to limited resources for research, the association between the expression of these genes and oocyte quality is not yet well understood. Studies have indicated that the expression of some genes will increase during the stages of oocyte maturity from GV to meiosis II (MII) (2, 3). This includes genes involved in growth and evolution of oocyte: growth differentiation factor 9 and bone morphogenetic protein 15 (GDF9 and BMP15); energy-producing mitochondrial gene: adenosine triphosphatase 6 (ATPase6); genes involved in meiosis advancement and formation of spindle apparatus: aurora kinase C, cell division cycle 25, cell division cycle 20, mitotic arrest deficient-like 1, budding uninhibited by benzimidazoles 1 (AURKC- CDC25- CDC20- MAD2L1- BUB1); and genes involved in DNA repair and cell cycle: breast cancer 1, Ataxia telangiectasia and rad3 related, Ataxia telangiectasia mutated (BRCA1- ATR- ATM) (2, 4). 

The oocyte cytoplasm transfer is a newly developed technique, which was first done in an animal study through direct injection and led to live birth (5-7). This technique has also been 43% successful in human studies, even in women with a repeated history of failed IVF due to poor cleavage fetal or embryonic fragmentation (8-11). The biological explanation for this technique is that the cytoplasm having some unknown factors could lead to the beginning or the activation of molecular development cascade in the recipient oocyte (12). 

The present study sought to evaluate the effect of transferring the cytoplasm of a mature oocyte to a GV oocyte (asynchronized cytoplasmic injection, in which the donor and recipient oocytes are not in the same cytoplasmic maturity stage) on nucleus and cytoplasmic maturity of the GV oocyte (as the recipient) at the mRNA levels. If the injection technique can enhance the expression level of genes involved in cell maturation by modifying the cytoplasm defects of the oocyte, it can be used as a complementary method with the 24-hour in vitro culture for maturation of GV oocytes and increasing the number of mature and efficient oocytes in infertile women.

## Materials and methods

Sixty six infertile couples who underwent IVF/ICSI between June 2012 and November 2013 at the Infertility Center of Tehran Women General Hospital, Tehran University of Medical Sciences, were included in this study. We included the women that had undergone IVF treatment because of male factor, tubal factor, or unexplained infertility. These women did not have ovulatory dysfunction, were aged ≤ 40 years, and had a normal baseline follicle stimulating hormone (FSH) and luteinizing hormone (LH) (< 10 mIU/mL). We obtained 120 GV oocytes donated by women. Normal GV oocytes were categorized into 3 groups randomly by the statistician, each consisting of 40 oocytes: the first comprised oocytes that did not receive the cytoplasm of mature oocytes; the second group comprised oocytes that did not receive the cytoplasm of mature oocytes but were incubated for 24 h; and the third group comprised oocytes that received 10-15% the cytoplasm of mature oocytes by a microinjection pipette and were then incubated for 24 h.

***Ovarian Stimulation***

The women underwent controlled ovarian stimulation (COS) with the gonadotropin releasing hormone agonist (GnRH-a) long protocol. The treatment started by administering oral contraception pill (OCP) on the 2^nd^ or the 3^rd^ day of the pervious menstrual cycle. The daily administration of Buserelin acetate 500 μg (Suprefact, Aventis, Germany) was started preceding the IVF cycle from day 21 until pituitary down-regulation (serum E2 < 50 pg/ml in the absence of follicular structures larger than 10 mm). The Buserelin dose was reduced to 250 μg/d until the day of human chorionic gonadotropin (hCG) injection. Ovarian stimulation was started on the 3^rd^ day of the current menstrual cycle by injection of rFSH Follitropin alfa (Gonal F, Serono, Italy) at a daily dose of 150 to 225 IU and was continued until hCG was injected. When at least 3 follicles with a mean diameter of 17 mm were developed (evaluated by transvaginal sonography), hCG 5000 IU /2/IM (Choriomon, IBSA Institut Biochimique S.A., Switzerland) was injected. About 34–36 h later, ultrasound-guided transvaginal oocyte retrieval was performed (13). All the women had mature oocytes for ICSI, but they donated immature ones (GV oocytes) to our study. 

***Oocyte Collection***

One twenty hundred morphologically normal GV oocytes were donated by 66 healthy women with normal ovarian reserve functions. The retrieved oocytes were collected in a Quinn’s Advantage Medium with HEPES (Sage, USA) supplemented with 20 % human serum albumin and then granulosa cells were removed from oocytes using mechanical and chemical (Hyaluronidase type 4, Sigma Aldrich, USA) methods under a stereomicroscope. After stripping off granulosa cells, we used an inverted microscope (Nikon, Tokyo, Japan) to monitor the maturity of the oocytes. Oocyte at the GV stage was characterized by enlarged nucleus and absent of polar body.

Then, the donated GV oocytes were categorized into three groups of forty; 1) control 1: 40 GV oocytes were collected and stored; 2) control 2: 40 GV oocytes were first incubated in 50 µl droplets of the culture G-1^TM^PLUS (Vitrolife, Sweeden) under mineral oil (Vitrolife, Sweeden) at 37 °C and 6% CO_2_ for in an incubator (Heraccel, Germany) 24 hr and then stored; and 3) intervention group: 10-15% of the cytoplasm was transferred from a mature oocyte into a GV oocyte using microinjection pipette (COOK, USA) under an inverted microscope (Nikon, Tokyo, Japan) in the contrast phase with a magnification of 200x, and then the oocytes were incubated for 24 hr under the same conditions as the control 2 group. Finally, each oocyte was individually transferred to RNase-free micro centrifuge tubes, and then RTL buffer (Amboin, Austin, USA) was poured on it. 

All samples were coded and kept in a refrigerator at a temperature of −80°C until the time of the analysis.

***Cytoplasm transfer in the intervention group***

First, the microinjection pipette (COOK, USA) was put under an inverted microscope (Nikon, Tokyo, Japan) in the contrast phase with a magnification of 200x. Then, a donor oocyte was held still under the microscope using the holder pipette (COOK, USA), and 7-14% (equal to the 750 µm length of the pipette tip to its arm) of its cytoplasm was gradually aspirated using the microinjection pipette. The cytoplasm was aspirated from the opposite pole of the polar body to preserve the nuclei of donor oocyte. Afterwards, the recipient GV oocyte was held constant under the microscope using the holder pipette. Some of its cytoplasm was first sucked into the microinjection pipette and then the whole cytoplasm from the donor oocyte was injected into the recipient oocyte (8). 

***RNA isolation, cDNA production and qPCR***

Studies have indicated that the expression levels of specific genes will increase during oocyte maturation from the GV stage to the MII stage. These genes include GDF9 and BMP15 (genes involved in growth and evolution of oocyte), ATPase6 (energy-producing mitochondrial gene), AURKC- CDC25- CDC20- MAD2L1- BUB1 (genes involved in meiosis advancement and formation of spindle apparatus), and BRCA1- ATR- ATM (genes involved in DNA repair and cell cycle). In our study, the expression levels of these genes were evaluated.

ALLELEID 6.0 software was used for designing Exon-Junction primers. Molecular evolutionary genetics analysis (MEGA 4) software was also used for conducting sequence alignment. Oligo 6 Software was employed for the final assessment (Temperature/ Formation/False priming sites). Finally, we assessed primers in NCBI BLAST, as presented in Table 1.

Before isolating the RNA, the GV oocytes were thawed within RLT buffer at room temperature and then pooled. To separate the RTL buffer from the pooled oocytes, they were then centrifuged at 12000 g for 3 minutes in order to extract total RNA, based on the standard protocol suggested by the manufacturer (Trizol, Invitrogen, USA). In order to remove genomic DNA contamination from the samples, the total RNA obtained from both groups was treated with DNase I (Fermentas, Sanktleon-rot, Germany). The total RNA concentration of the pooled germinal vesicle oocytes after treatment was 594 μg/ml for the first control group, 672 μg/ml for the second control group, and 615 μg/ml for the intervention group, determined by a Thermo Scientific Nano Drop 2000 Spectrophotometer. cDNA was synthesized according to manufacturer's instructions (Fermentas, Sanktleon-rot, Germany) using random hexamer primers.

**Table 1:** Oligonucleotide primer sequences used for qPCR in the present study

**Gene name**	**Accession no.**	**Primer**	**Product Size (bp)**
GDF9	NM_005260.4		
Sense		CCAGGTAACAGGAATCCTTC	162
Antisense		GGCTCCTTTATCATTAGATTG	
BM15	NM_005448.2		129
Sense		CCTCACAGAGGTATCTGGC	
Antisense		GGAGAGATTGAAGCGAGTTAG	
ATPase 6	YP_003024031.1		123
Sense		CTGTTCGCTTCATTCATTG	
Antisense		GGTGGTGATTAGTCGGTTG	
NAIP	NM_004536.2		184
Sense		GGAGTATTTGGATGACAGAAAC	
Antisense		TAGATTACCACTGGAGTCTTCC	
TP53	NM_000546.5		184
Sense		GGAGTATTTGGATGACAGAAAC	
Antisense		TAGATTACCACTGGAGTCTTCC	
BUB1	NM_001278616.1		100
Sense		AAGGTCCGAGGTTAATCC	
Antisense		CACTGGTGTCTGCTGATAGG	
MADL2	NM_002358.3		169
Sense		CTTCTCATTCGGCATCAAC	
Antisense		ACACTTGTATAACCAATCTTTCAG	
CDC20	NM_001255.2		202
Sense		GATGTAGAGGAAGCCAAGATC	
Antisense		CCACAAGGTTCAGGTAATAGTC	
ATR	NM_001184.3		150
Sense		GATGCCACTGCTTGTTATG	
Antisense		CCACTCGGACCTGTTAGC	
ATM	NM_000051.3		107
Sense		GCATTACGGGTGTTGAAG	
Antisense		ATATAGAAGGACCTCTACAATG	
BRCA1	NM_007294.3		141
Sense		CACTCAGCAGAGGGATACC	
Antisense		TCAAGGGCAGAAGAGTCAC	
AURKC	NM_001015878.1		176
Sense		CGCACAGCCACGATAATAG	
Antisense		CACATTGTCTTCCTCCTCAG	
CDC25A	NM_001789.2		96
Sense		CTTTATGAAATGCCAGTCTTAC	
Antisense		CTCTTGGTGCGGAACTTC	
CDC25B	NM_001287516.1		120
Sense		TGACTTAAAGGATGATGATGC	
Antisense		CGCTGGCACTTGCTGTAC	
β.actin	NM_001101.3		90
Sense		CAAGATCATTGCTCCTCCTG	
Antisense		ATCCACATCTGCTGGAAGG	

We performed qPCR on the cDNA obtained from the pooled of GV oocytes. Relative gene expression was calculated as the abundance ratio of each target gene to β-actin.

Quantitative real time PCR reactions were carried out in triplicates using an ABI Prism 7300 Sequence Detector (Applied Biosystems, foster, USA) in a total volume of 20 μl containing 250 ng cDNA, 5 pmol gene specific primers and SYBR Green reagent (Applied Biosystems). The protocol for qPCR was initiated with a denaturizing step at 95°C for 30 seconds, followed by 50 cycles of 2-step, real-time PCR under the following conditions: 5 seconds at 95°C for denaturation and 30 seconds at 59–60°C for annealing and extension. No template control (NTC) was used as the negative control. The specificity of the PCR fragments was determined using melting curve analysis. All melting curves produced one peak for each of the PCR products.

***Ethical considerations***

The present study was approved by the ethics committee of Tehran University of Medical Sciences. The study was completely explained to the women, and informed consent was obtained before collecting germinal vesicles oocytes. The mature oocytes used in the present study were also donated by the women participating in the study. The study was officially registered with the following code: IRCT 2013090512307N1.

***Statistical analysis***

We used One-way ANOVA to compare quantitative variables between the two groups and chi-square for qualitative variables by SPSS version 16 (Chicago, IL, USA). The significance level was set at 0.05. The efficiency values given by the Linreg software and relative expression were calculated using the REST 2009 software (Qiagen, Hilden, Germany), which is a standalone software tool used for estimating up and down regulation for gene expression studies (14). The ΔΔCT was obtained by finding the difference between the groups. The fold change was calculated as FC = 2^-ΔΔCT^. For this purpose, β.actin was used as the reference gene for expression normalization.

## Results

There were no significant differences in the age, hormonal profile, number of oocytes retrieved, infertility duration, type and cause of infertility among the three groups (p > 0.05), as presented in Table 2 and Table 3.

**Figure 1:**
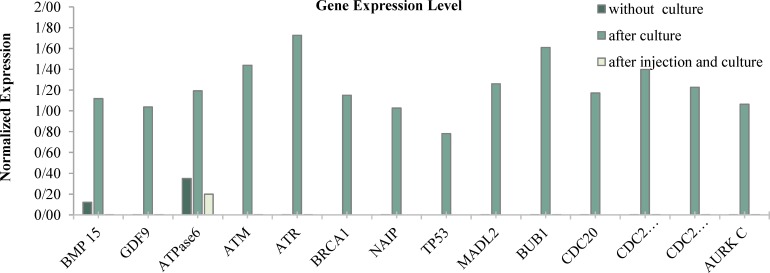
A comparative assessment of the gene expression level among the control 1, control 2, and intervention groups measured using the real time PCR method

The present study showed that the expression levels of genes involved in the cytoplasmic maturity (GDF9, BMP15), and energy-producing mitochondria gene (ATPase6) were significantly higher in the pooled oocytes of 2^nd^ control group than those of the 1^st^ control and intervention groups (p < 0.001). The genes involved in the meiosis (CDC25, AURKC), spindle check point (BUB1,CDC20, MAD2L1), DNA repairing and cell cycle checkpoint (ATR, ATM, BRCA1) did not have any expression in the 1^st ^and intervention groups; however, these genes were expressed in the 2^nd^ group, significantly. In the second group, the highest expression level was observed for genes involved in the DNA repairing and cell cycle checkpoint. On the other hand, in the third group, none of the genes were expressed, except for ATPase 6; even in this case, the expression level of this gene in this group of oocytes was significantly lower than that in other groups (p < 0.001), as presented in Figure 1 and Table 4. After 24 h, based on the morphology of the oocytes, meiosis assumption was significantly higher in the third group than in the second group (95% vs. 68%, p < 0.001).

**Table 2:** Mean (standard deviation) age, duration of infertility, number of oocytes retrieved, serum LH, FSH, TSH, PRL, AMH, and serum 17-beta estradiol in the control 1, control 2, and intervention groups

**Variable**	**Control 1** **(n = 24)**	**Control 2** **(n = 22)**	**Intervention** **(n = 20)**	**Total** **(n = 66)**	**P value** **One-Way ** **ANOVA Test**
Age (years)	31.7 ± 5.1	30.6 ± 5	30.2 ± 5.4	30.9 ± 5.1	0.575
Duration of infertility (years)	6.4 ± 3.2	4.4 ± 2.4	5.6 ± 2.8	5.5 ± 2.9	0.064
Number of oocytes retrieved	11.7 ± 4.3	11.9 ± 5.4	11.1 ± 4.6	11.6 ± 4.7	0.833
MII	7.0 ± 2.9	7.8 ± 3.5	6.9 ± 2.9	7.2 ± 3.1	0.593
MI	2.2 ± 1.8	1.5 ± 1.5	1.5 ± 1.7	1.8 ± 1.7	0.231
GV	2.1 ± 1.6	2.4 ± 2.3	2.5 ± 1.6	2.3 ± 1.8	0.736
Serum 17 β-estradiol*(Pg/ml)	3666.7 ± 2475.2	3782.4 ± 2091.8	4380.2 ± 3270.0	3928.1 ± 2630.4	0.649
Serum LH (IU/L)	5.06 ± 2.9	7.3 ±4.6	5.4 ± 2.4	5.9 ± 3.5	0.080
Serum FSH (IU/L)	6.2 ± 2.4	6.9 ± 2.1	6.2 ± 1.8	6.4 ±2.1	0.466
Serum TSH (μIU/L)	2.6 ± 2.0	1.9 ± 1.0	2.2 ± 1.0	2.3 ±1.4	0.385
Serum PRL (ng/ml)	124.3 ± 173.7	226.2 ± 283.7	78.7 ± 141.5	145.5 ±215.9	0.075
Serum AMH (ng/ml)	5.6 ± 5.7	3.8 ± 3.5	7.3 ±6.9	5.6 ±5.7	0.175

* On the day of hCG administration; LH: luteinizing hormone; FSH: follicle-stimulating hormone; TSH: thyroid-stimulating hormone; PRL: prolactin; AMH: anti-mullerian hormone; MII: mature oocyte (Meiosis II); MI: immature oocyte (Meiosis I); GV: Germinal Vesicle.

**Table 3:** Distribution of the causes of infertility in the control 1, control 2, and intervention groups

**Variable**	**Control 1 (%)**	**Control 2 (%)**	**Intervention (%)**	**P value** **Pearson Chi –Square**
Cause of infertility				0.915
Male factor	13 (54.2)	10 (45.5)	9 (45.0)	
Tubal factor	9 (37.5)	10 (45.5)	8 (40.0)	
Unexplained	2 (8.3)	2 (9.1)	3 (15.0)	
Total	24.0 (100.0)	22.0 (100.0)	20.0 (100.0)	
Type of infertility				
Primary	21 (87.5)	13 (59.1)	16 (80.0)	
Secondary	3 (12.5)	9 (40.9)	4 (20.0)	
Total	24.0 (100.0)	22.0 (100.0)	20.0 (100.0)	0.070

Figure 1 illustrates the relative expression of genes in the pool of oocytes in the control 1 (without incubation), control 2 (incubated), and intervention (cytoplasmic transfer) groups. In the pooled oocytes of the control 1 group, only the BMP15 and ATPase6 were expressed. In the pooled oocytes of the control 2 group, all of the studies genes were expressed; however, it is noteworthy that the genes involved in DNA repair and spindle apparatus formation were expressed significantly more than apoptotic and anti-apoptotic genes (post-hoc P-value of 0.041 and 0.048, respectively). In the pooled oocytes of the intervention group, only the ATPase6 was expressed. The b-actin gene was considered as the internal control.

## Discussion

The influential role of the oocyte cytoplasm in the evolution and implantation of the embryo is not a new concept. Impaired performance of the oocyte cytoplasm will lead to failure in fertilization, implantation, fetal development, and pregnancy. Additionally, immature cytoplasm can have a negative effect on the male pronucleus and reduce the amount of implantation (1). Theoretically, transferring the cytoplasm of a mature oocyte to a GV oocyte (due to cytoplasmic defects) can lead to the completion of growth cycle in the recipient oocyte, mainly because the donor mature oocyte possesses factors regulating the cell cycle, healthy mitochondria, microtubules involved in cell division, enzymes involved in DNA replication, and increased level of mRNA (12). 

Considering the successful transfer of cytoplasm from a mature oocyte to high-risk oocytes (due to cytoplasmic defects) in both animal and human studies (8-11), the present work attempted to evaluate the effect of transferring the cytoplasm of a mature oocyte to a GV oocyte (asynchronized cytoplasmic injection, in which the donor and recipient oocytes are not in the same cytoplasmic maturity stage) on nucleus and cytoplasmic maturity of the GV oocyte (as the recipient) at the mRNA and morphological transformation levels.

**Table 4:** The genes expression of oocyte in control 1, control 2, and intervention groups

**Gene symbol**	**Gene title**	**Control 1**		**Control 2**		**Intervention**	
		**Exist**	**Not ** **exist**	**Exist**	**Not ** **exist**	**Exist**	**Not ** **exist**
Transforming growth factorsβ							
GDF9	Growth differentiation factor 9		+	+			+
BMP15	Bone morphogenetic protein 15	+		+			+
Mitochondria							
ATPase6	Adenosine triphosphatase 6	+		+		+	
Anti/ Pro apoptotic							
NAIP	Neuronal apoptosis inhibitory protein		+	+			+
Tp53	Tumor protein p53		+	+			+
Cell cycle checkpoint markers							
BUB1	Budding uninhibited by benzimidazoles 1		+	+			+
MAD2L1	Mitotic arrest deficient-like 1		+	+			+
CDC20	Cell division cycle 20		+	+			+
Meiosis							
AURK C	Aurora kinase C		+	+			+
CDC25A	Cell division cycle A		+	+			+
CDC25B	Cell division cycle B		+	+			+
DNA repair markers							
ATR	Ataxia telangiectasia and Rad3		+	+			+
ATM	Ataxia telangiectasia mutated		+	+			+
BRCA1	Breast cancer 1		+	+			+
Reference gene							
B.actin	Beta actin	+		+		+	

Healthy cytoplasm mitochondria have a critical role in the development of mediation. The ATPase6 is an indicator of oocyte maturation, and its production rate is dependent upon the mitochondrial performance of the cell. This gene plays a critical role in oocyte maturation and embryonic cleavage (15-17). As mentioned in the results section, there was 54% decrease in the expression level of this gene in the intervention group compared to the other two control groups, suggesting that following asynchronized cytoplasmic transfer, the mitochondria from the mature oocyte has been removed or disturbed after entering the GV oocyte, similar to the removal of sperm mitochondria after entering the egg (18). 

Lack of expression of AURK C and CDC25 genes, which are involved in advancement of meiosis, was expected in the GV oocytes of the control 1 group. However, these genes exhibited increased expression levels in the control 2 group; suggesting that meiosis improves following a 24-hr incubation period. The occurrence of germinal vesicle break down (GVBD) might have occurred due to the CDC25B phosphatase effect and AURK C gene expression (19). In the intervention group, however, these two genes were not expressed. BMP15 and GDF9, which are involved in the growth and evolution of the oocyte (20-24), also exhibited similar patters. 

In the intervention group, the expression of CDC20, MAD2L1, and BUB1, which are involved in the formation of cytoskeleton, was disturbed; this might have been observed due to the rupture of cytoskeletons (2, 25). On the other hand, in the control 2 group, the expression level of these genes increased, and as Spindle assembly checkpoint, led to the formation of spindle apparatus. TP54, ATR, ATM, and BRCA1 proteins, which are involved in DNA repair and cell cycle, act as checkpoints in the interphase stage of the cell cycle (2, 4, 26, 27). These proteins prevent meiosis until DNA replication is complete in the interphase stage of the cell cycle. Additionally, the TP53 protein prevents the meiosis until DNA repair of the cell is complete. The BRCA1 regulates the TP53 under stress, meaning that TP53 will initiate the DNA repair pathway instead of initiating the apoptotic pathway (3, 4). In the present work, BRCA1, ATR, and ATM had increased expression levels compared to other genes in the control 2 group, indicating damage to the nucleus DNA in the GV oocytes. The higher expression level of BRCA1 compared to that of TP53 in the control 2 group suggests that the TP53 protein has initiated DNA repair pathway instead of the apoptotic pathway. Nonetheless, these genes were not expressed in the intervention group.

The neuronal apoptosis inhibitory protein (NAIP) exhibited increased expression level in the control 2 group. NAIP is a member of the Inhibitor of Apoptosis Proteins family and can cause to the evolution of primary follicle to follicle Graaf during folliculogenesis and indirectly lead to increased viability of the oocyte through inhibition of apoptotic activities of caspases 3 and 9 (28). In the present work, for the first time, we showed the expression of this gene in human oocyte. 

Although mature oocyte cytoplasm transfer techniques to GV oocytes can morphologically enhance the resumption of meiotic maturation to levels as high as 95% in the recipient oocyte, this disrupts the expression of genes involved in the cytoplasmic maturity of the cell. The disturbed expression of genes in the intervention group compared to the control groups can be attributed to impaired Ca^2+^ oscillation and intra cell signaling, interference in the activity of transcription factors, insufficient transfer of mRNA (2, 3), asynchronized cytoplasmic transfer, removal of the  mitochondria transferred to the GV oocyte (18), disturbed epigenetic regulation (29), or nuclear DNA deficiency in the GV oocyte (based on the observed increase in the expression of genes involved in DNA repair in the nuclei). Although the results of previous studies have shown that the cytoplasm transfer from a mature oocyte to a high-risk oocyte with cytoplasmic defects may lead to restoring the normal growth of the recipient oocyte, this study suggests, by evaluating the mRNA, that the cytoplasm transfer technique is not effective in cytoplasmic maturity of the recipient GV oocytes. In contrast, 24-hr in-vitro culture is associated with increased expression of genes involved in the cytoplasmic maturity, meiosis, spindle and cell cycle checkpoint in GV oocytes.
